# Generalizing across moral sub-domains: infants bidirectionally link fairness and unfairness to helping and hindering

**DOI:** 10.3389/fpsyg.2023.1213409

**Published:** 2023-07-20

**Authors:** Inderpreet K. Gill, Jessica A. Sommerville

**Affiliations:** Department of Psychology, University of Toronto, Toronto, ON, Canada

**Keywords:** fairness, infants (birth to 2 years), moral development, moral judgment, trait inference

## Abstract

Across two experiments, we investigated whether infants use prior behavior to form expectations about future behavior within the moral domain, focusing on the sub-domains of fairness and help/harm. In Experiment 1, 14- to 27-month-old infants were familiarized to an agent who either helped or hindered another agent to obtain her goal. At test, infants saw the helper or hinderer perform either a fair or unfair distribution of resources to two recipients. Infants familiarized to helping looked longer to the unfair distribution than the fair distribution at test, whereas infants familiarized to hindering looked equally at both test events, suggesting that hindering led infants to suspend baseline expectations of fairness. In Experiment 2, infants saw these events in reverse. Following familiarization to fair behavior, infants looked equally to helping and hindering; in contrast, following familiarization to unfair behavior, infants looked significantly longer to helping than hindering on test, suggesting that prior unfair behavior led infants to expect the agent to hinder another agent’s goals. These results suggest that infants utilize prior information from one moral sub-domain to form expectations of how an individual will behave in another sub-domain, and that this tendency seems to manifest more strongly when infants initially see hindering and unfair distributions than when they see helping and fair distributions. Together, these findings provide evidence for consilience within the moral domain, starting by at least the second year of life.

## Introduction

The ability to use an individual’s behavior in one context to make inferences about how that individual will behave in a different context (Wernimont and Campbell, 1968; Lammers et al., 2017), provides a basis for coordinating our actions with other people, and enables us to make decisions regarding whom to approach and whom to avoid. One means by which (Western) adults predict others’ future behavior is through the rapid attribution of traits based on limited behavioral evidence (Lupfer et al., 2000; Uleman et al., 2008; Shimizu, 2012). The tendency to infer morally relevant traits, in particular, is prevalent in adults, as an individual’s moral character may have consequences for one’s own welfare (Goodwin et al., 2014). At present the earliest developmental origins of this ability is relatively uncharted, given debates concerning the nature of young children’s moral understanding (see Killen and Smetana, 2015 for a review), and the emergence of trait reasoning in childhood (see Heyman, 2009 for a review). Here we investigate the developmental origins of the ability to use information from one moral sub-domain to make inferences about how an individual will behave in another sub-domain in infancy, focusing on fairness and help/harm given their prominence in theories of morality (Ting et al., 2019; Decety et al., 2021).

### The developmental origins of children’s trait inferences

Research indicates that children’s ability to make trait inferences, broadly construed, varies considerably according to the experimental approach and task requirements. Initial work suggested that it is not until mid-childhood that children reason about others’ traits: children first describe others’ behaviors using trait terms at about age 8 (Livesley and Bromley, 1973; Peevers and Secord, 1973; Heyman and Gelman, 1999), and whereas 9- and 10-year-old children expect behavioral consistency across contexts, younger children do not (Rholes and Ruble, 1984; Kalish, 2002). Subsequent studies, however, demonstrated that children as young as 4 infer traits when multiple exemplars of an initial behavior are provided (Heller and Berndt, 1981; Cain et al., 1997; Boseovski and Lee, 2006), when children are told about two different actors that act in opposing directions (e.g., generous versus selfish; Heller and Berndt, 1981; Cain et al., 1997), or when they are asked to identify a trait from a given behavior, or use a trait to infer a subsequent behavior (Heyman and Gelman, 1999; Liu et al., 2007).

Moral trait inferences may emerge before the preschool years given the importance of this tendency in everyday social interactions (i.e., Rakoczy et al., 2016). Indeed, moral trait terms are some of the first that are utilized in children’s language output (Yuill, 1992; see Franchin et al., 2019): as early as 19–22 months, infants use terms such as ‘good,’ ‘bad,’ ‘naughty,’ and ‘nice’ (Bloom et al., 1975; Fenson et al., 1994), and by 30 months children start to apply words like ‘good’ and ‘bad’ to moral contexts (Snow, 1987). These findings raise the possibility that young children may make moral trait inferences, even if they struggle to form trait inferences for non-moral traits.

### Moral sensitivities in infancy

Recent work suggests that infants possess moral sensitivities in the sub-domains of fairness and help/harm. Infants expect other agents to approach those that previously helped versus those that harmed (Kuhlmeier et al., 2003), and prefer agents that help over agents that hinder in third-party settings by 6 months of age (Hamlin et al., 2007; Hamlin and Wynn, 2011; Hamlin, 2015). By roughly 10 months of age (or earlier, when distributions featuring 2:0 versus 1:1 are used; see Buyukozer Dawkins et al., 2019) infants show a nascent sensitivity to fairness, expecting equal distributions (Meristo et al., 2016; Ziv and Sommerville, 2017), and by 13 months of age, evaluate others based on their distributive behavior, showing preferences for those that distribute resources equally over those that do so unequally (Burns and Sommerville, 2014), and link fair and unfair behavior to positive and negative stimuli, respectively (DesChamps et al., 2016; see also Geraci and Surian, 2023). Infants’ sensitivity to equal distributions can also be seen in cases where a distributor intended to perform an equal distribution but failed: 9-month-olds prefer a distributor that tried to divide resources equally but failed to a distributor who tried but failed to divide resources unequally (Geraci et al., 2022; but also see Strid and Meristo, 2020).

Of course, mature moral understanding entails a recognition that behavior from one moral sub-domain may have consequences for how an individual will act in another sub-domain; adults may expect a stranger who returns their dropped wallet to also hold the door open for them as they exit the bus. In a recent study, Gill et al. (under review)^1^ demonstrated that children aged 4- to 7 show similar tendencies: children reported greater surprise to fair (versus unfair behavior) after a protagonist hindered versus helped another individual. In addition, children reported greater surprise to the protagonist helping (versus hindering) another person after she previously distributed resources unfairly versus fairly. While children generalize from help/harm to fairness, and from fairness to help/harm, they did so uniquely from negative behavior: children’s surprise reports following fair behavior or helping behavior did not vary based on the test event. Thus, by age 4 children generalize across moral sub-domains after seeing moral transgressions.

This generalization tendency may extend to infants. Surian and colleagues (Surian et al., 2018) investigated infants’ ability to engage in moral generalization from help/harm to fairness (see also Ting and Baillargeon, 2021) by familiarizing 14-month-old infants to a protagonist helping another agent by pushing them up a hill or hindering the agent by pushing them down a hill. On test, infants in both conditions saw the previously helpful/hindering protagonist distribute two strawberries to two recipients equally (1:1) or unequally (0:2). After seeing the protagonist help another agent, infants looked longer to unfair than fair behavior. However, after seeing the protagonist hinder another agent, infants looked equally to fair and unfair behavior. These findings suggest that infants suspend expectations for fair behavior after learning that an agent hindered another agent’s goals.

In the current study, we investigated 14- to 27-month-old infants’ tendency to generalize across the sub-domains of fairness and help/harm. We first sought to conduct a conceptual replication of Surian et al. (2018): after seeing an actor help or hinder another individual, infants saw that actor perform equal or unequal resource distributions and their looking was recorded. Our study differed from Surian et al. (2018) in three ways. First, we used real-world actors rather than animations; a demonstration that infants show similar patterns documented by Surian et al. (2018) with real-world actors would provide increased confidence that infants apply such generalization tendencies in everyday life. Second, we used different helping and hindering events, modeled after Hamlin and Wynn (2011) and Hamlin (2015), to provide increased confidence that the results generalize across multiple instances of helping/hindering. Third, and perhaps most critically, our distribution events featured 5:1 versus 3:3 distributions, rather than 2:0 versus 1:1 distributions given concerns that 2:0 distributions may conflate social exclusion with unfairness because the actor who receives no resources is not included in the exchange (see DesChamps et al., 2016). As in Surian et al. (2018), we predicted that infants shown helping will look longer to unfair versus fair events, but that these expectations would be suspended following hindering leading infants to look equally at the fair versus unfair events.

In Experiment 2, infants watched these events in reverse: they saw fair or unfair distributions followed by helping or hindering behavior. Thus, we investigated whether infants possess a tendency to generalize across fairness and help/harm in a bi-directional manner. We also sought to compare the relative strength of these generalizations based on the original sub-domain. On the one hand, one might predict that infants will generalize more strongly from hindering versus unfairness given that hindering is often see as more egregious than unfairness (Yucel et al., 2022), and that infants appear to be sensitive to help/harm before fairness/unfairness in their evaluations (Sommerville, 2022). On the other hand, we might expect the opposing pattern given that some work suggests that while infants have baseline expectations for fair distributions, they do not have baseline expectations for helpful behavior (but see Hamlin et al., 2007; Lucca et al., 2018).

## Experiment 1

### Method

#### Participants

The final sample consisted of 56 14- to 27-month-old infants (age range: 14 months 2 days to 27 months; *M* = 17 months 28 days; 33 female, 23 male). Participants were recruited from an online database maintained by a large university in North America. Our sample^2^ consisted of 42% White, 19% Biracial (i.e., Black/Indigenous and/or Metis, East Asian/White, Latin, Central or South American/White, South Asian/White, or West Asian/White), 12% East Asian, 9% South Asian, 6% Southeast Asian, 3% Asian Indian, and 3% Other. The data of 23 additional participants was excluded due to fuss out (*n* = 2), inattentive child (*n* = 4), procedural errors (*n* = 3), technical errors (*n* = 3) or other errors (i.e., parental interference, environmental interference; *n* = 11). Parental consent was obtained on behalf of all the infants through a Qualtrics survey parents completed prior to testing.

#### Materials

PowerPoint presentations were used to display pre-recorded videos for the tasks. Infants’ looking was measured online via Zoom by the experimenter using jHab (Casstevens, 2007), a computer-based program that allows researchers to measure duration looking.

#### Procedure

The experiment took place over Zoom and was presented to infants through a PowerPoint presentation screensharing. The experimenter guided parents in turning off ‘side-by-side’ mode in Zoom, hiding the infants’ self-view and the experimenter’s video on their end so that infants only saw the PowerPoint slides. Parents had their eyes closed and/or covered. They were told to remain silent and neutral throughout the session; compliance was monitored by the primary experimenter.

The experiment utilized a violation of expectation (VOE) paradigm. Infants watched a series of familiarization trials and then a test event, and their duration attention to the outcome of the events were recorded.

##### Familiarization preview

Before familiarization, infants were shown a video of an agent struggling to open a translucent box to retrieve a toy, to ensure that infants appreciated that the agent had the goal of opening the box to get the toy inside.

##### Familiarization

Infants were randomly assigned to the helping condition or hindering condition. They watched a total of four 15-s-long familiarization trials.^3^ Infants’ duration looking was recorded from the end of the familiarization trial (to the still screen image) until they looked away for 2 s or 30 s elapsed.

Across both conditions, infants saw the agent struggle to open the box two times. In the helping condition, on her third try, the protagonist reached over and lifted the lid on the side of the box closest to her, enabling the agent to retrieve the toy. In hindering condition, on her third try, the agent lifted the lid such that the box was half-way open, and the protagonist then reached over and slammed the lid of the box down preventing the agent from retrieving the toy.

##### Test trials

Infants were randomly assigned to the fair or unfair test event (between subjects).

Both test events featured the same protagonist from the familiarization trials, and two recipients, sitting at a table. In the fair test event. The protagonist held up a bowl of cookies and said, “Mmm, yummy.” The other two actors, after seeing the cookies, said “please” and moved their empty plates forward toward the protagonist at the same time. The protagonist then preceded to distribute the cookies fairly (e.g., giving an equal number of cookies to each recipient) saying “here” as she placed the cookies on each plate. At the end of the distribution she said, “There! All done.” The unfair test event was identical except that in this event one actor received 5 cookies and the other actor received only 1 cookie. Infants saw a single test event: either the fair test event or the unfair test event.

##### Reliability coding

Infants looking was coded online using jHab (Casstevens, 2007) by an experimenter and a secondary coder, unaware of which condition a participant was run in, coded infants’ looking time. The secondary coder reliability coded 33% of the total sample.^4^ The original coder and secondary coder’s looking times were highly correlated on familiarization trials, *r* = 0.995, *p* < 0.001, and on test trial, *r* = 0.982, *p* < 0.001.

### Results

Across both experiments, we adopted the same analytic strategy. First, we analyzed for effects of familiarization trial type and familiarization trial number, to determine whether, in the current context, infants showed differential attention to the familiarization trials (which could account for differential attention to the test events), and to ensure that infants’ attention was declining across familiarization trials, indicating that they were encoding the familiarization events. Separate ANOVAS for each familiarization type were then conducted to ensure that infants’ attention declined equally for each familiarization type.

We next conducted omnibus analyses on looking on test trials as a function of familiarization type and test trial and their interaction. We conducted planned comparisons of looking to each test trial type, split by familiarization type, regardless of the outcome of the omnibus analyses, to provide direct comparisons across studies.

#### Familiarization trials

**Figure 1:**
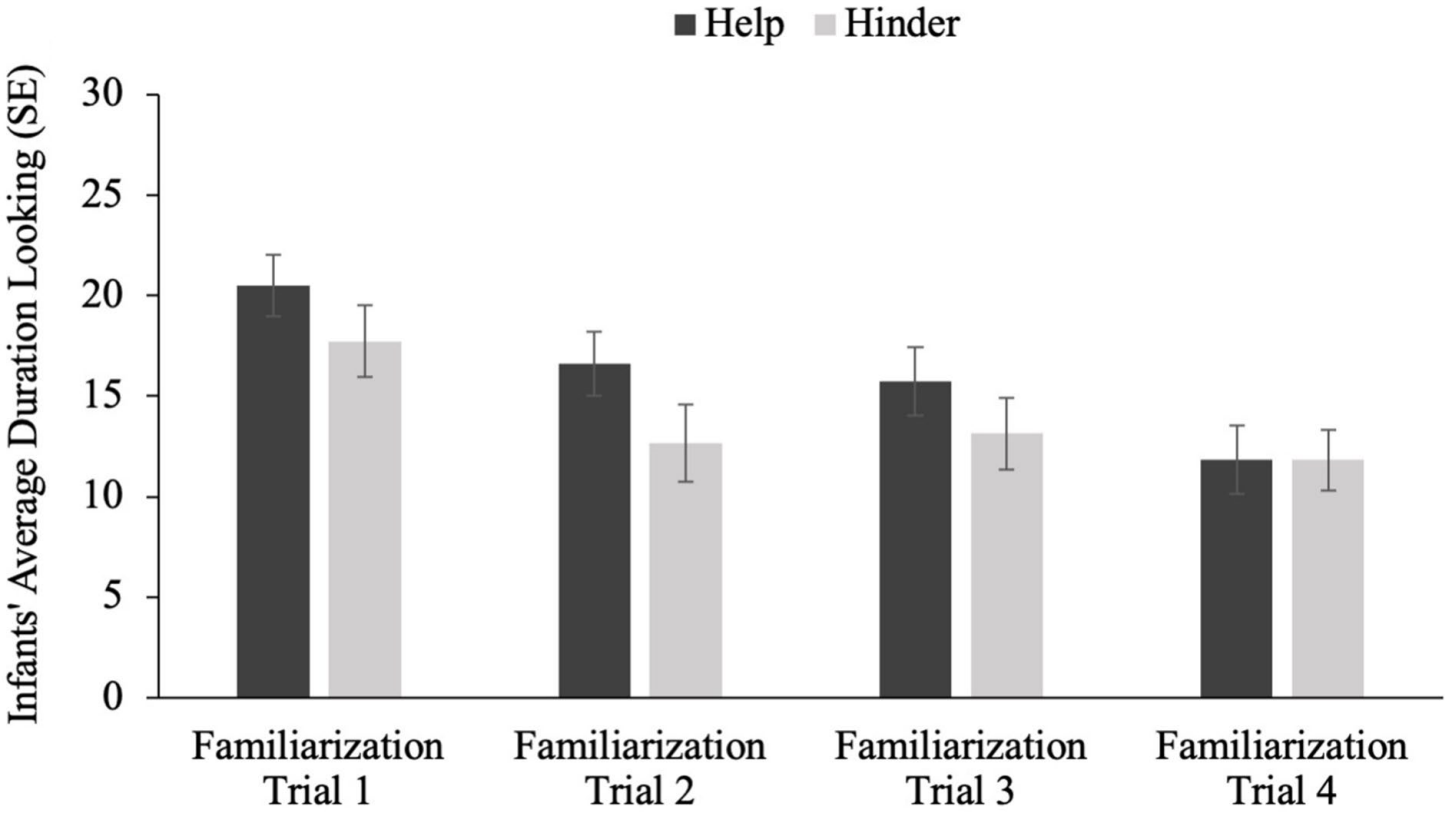
Average looking time on each familiarization trial number by familiarization trial type.

A 2 (Familiarization Type: help vs. hinder) × 4 (Familiarization Trial Number) ANOVA revealed a significant main effect of familiarization trial number (*F* (3, 53) = 9.766, *p* < 0.001), but no effect of familiarization type [*F*(1, 55) = 2.381, *p* = 0.129] and no interaction between familiarization type and trial number [*F*(3, 53) = 1.008, *p* = 0.391]. Infants’ average looking time decreased with each familiarization trial irrespective of the familiarization type (see Figure 1^5^).

Two separate repeated measures ANOVAs, with Familiarization Trial Number as a within-subject factor, demonstrated that infants’ looking times significantly declined for both the helping familiarization trials [*F*(1, 3) = 7.472, *p* < 0.001] and the hindering familiarization trials [*F*(1, 3) = 3.495, *p* = 0.019].

#### Test trials

**Figure 2:**
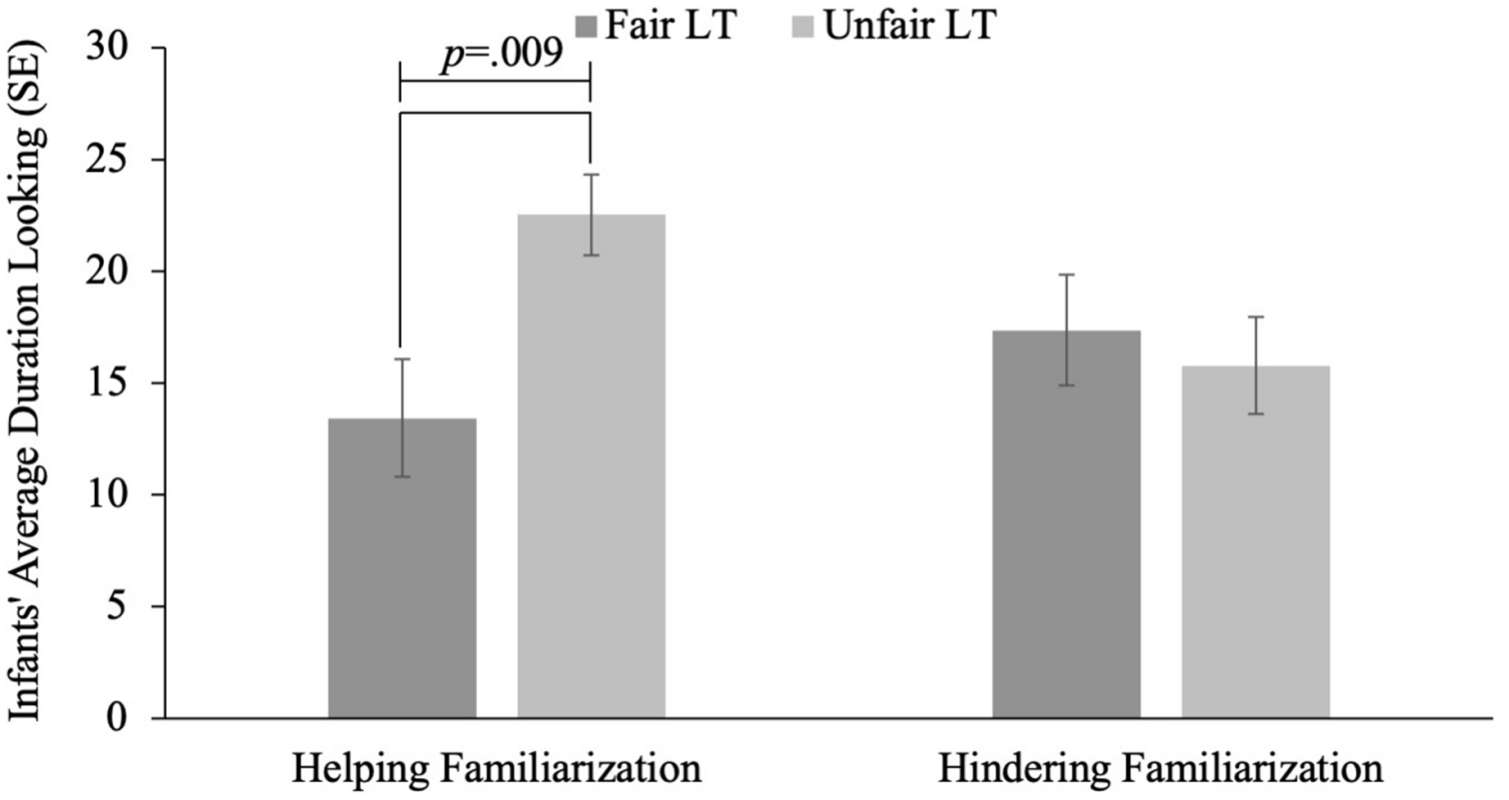
Infants’ average looking time to fair and unfair distributions following helping and hindering behavior.

An ANOVA looking at infants’ looking time at test as a function of familiarization and test trial, indicated no main effects of familiarization, *F*(1, 55) = 0.337, *p* = 0.542, or test trial, *F*(1, 55) = 2.662, *p* = 0.109. Critically, a significant interaction between familiarization and test trial was obtained, *F*(1, 55) = 5.373, *p* = 0.024; see Figure 2.

An independent samples t-test revealed that infants who were familiarized to helping behavior looked longer at the unfair distribution (*M* = 22.53, *SE* = 1.83) versus the fair distribution (*M* = 13.45, *SE* = 2.65), indicating they were surprised to observe the helper subsequently distributing resources unequally, *t*(26) = 2.82, *p* = 0.009, *d* = 1.067. In comparison, infants who were familiarized to the hindering condition looked equally to the unfair (*M* = 15.79, *SE* = 2.17), and fair (*M* = 17.37, *SE* = 2.47) distributions, *t*(26) = 0.48, *p* = 0.635, *d* = 0.182, suggesting that following hindering infants suspended their baseline expectations for fair resource distributions.

## Experiment 2

In Experiment 1, consistent with Surian et al. (2018), we found that infants who were familiarized to helping behavior were surprised when the helper subsequently distributed resources unfairly, whereas infants who were familiarized to hindering behavior suspended expectations for fair or unfair distributions. In Experiment 2, we reversed the direction of moral behavior to investigate if infants generalize from fairness/unfairness to help/hinder, to determine whether infants make the link between the sub-domains of help/harm and fairness in a bi-directional manner.

### Method

#### Participants

The final sample consisted of 56 14- to 26-month-old infants (age range: 14 months 5 days to 26 months 15 days; *M* = 18 months 9 days; 31 female, 25 male). Participants were recruited from an online database. Our sample^6^ consisted of 56% White, 18% Biracial (i.e., East Asian/Southeast Asian, East Asian/Indo-Caribbean, East Asian/White, Southeast Asian/White or South Asian/White), 8% East Asian, 5% Multi-Racial (i.e., East Asian/Jewish/Eastern European) or South Asian/Southeast Asian/White, 5% Arab, 2% Black, and 2% Caribbean/West Indian. The data of 23 additional participants was excluded due to fuss out (*n* = 4), inattentive child (*n* = 6), procedural errors (*n* = 1), technical errors (*n* = 1) or other errors (i.e., parental interference, environmental interference; *n* = 11). Parental consent was obtained on behalf of all the infants through a Qualtrics survey parents completed prior to testing.

#### Procedure

Infants participated in the same procedure as in Experiment 1, with the exception that the events were reversed: infants saw familiarization trials (fair or unfair distributions), a preview trial (actor struggling to open a box), and a test event (either helping or hindering).

##### Reliability coding

Infants looking was coded online using jHab (Casstevens, 2007) by an experimenter and a secondary coder, unaware of which condition a participant was run in, coded infants’ looking time. The secondary coder reliability coded 98% of the total sample.^7^ The original coder and secondary coder’s looking times were highly correlated on familiarization trials, *r* = 0.968, *p* < 0.001, and on test trials, *r* = 0.985, *p* < 0.001.

### Results

#### Familiarization trials

**Figure 3:**
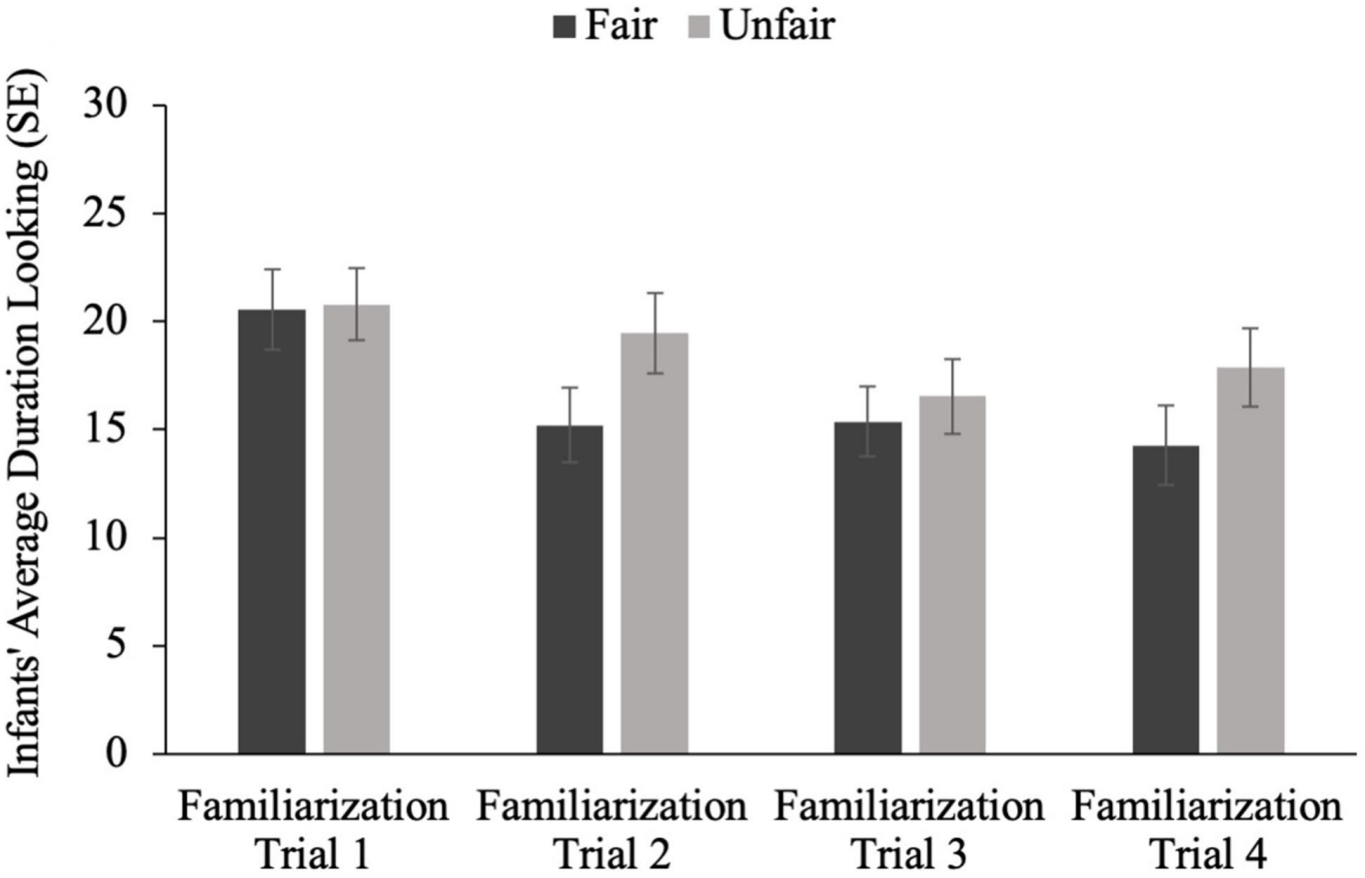
Average looking time on each familiarization trial number by familiarization trial type.

A 2 (Familiarization Type: fair distribution vs. unfair distribution) × 4 (Familiarization Trial Number) ANOVA revealed a main effect of familiarization trial number [*F*(3, 53) = 5.412, *p* = 0.001], but no effect of familiarization condition [*F*(1, 55) = 1.529, *p* = 0.222] and no significant interaction [*F*(3, 53) = 1.027, *p* = 0.382]; see Figure 3.

Two separate repeated measures ANOVAs, with Familiarization Trial Number as a within-subjects factor, demonstrated that while infants’ looking significantly declined for the fair distribution [*F*(1, 3) = 5.131, *p* = 0.003] it did not for the unfair distribution [*F*(1, 3) = 1.723, *p* = 0.169]. These findings suggest that although infants did not look longer overall at the unfair familiarization event, as might be expected by prior work (i.e., Geraci and Surian, 2011; Schmidt and Sommerville, 2011; Sloane et al., 2012), there is some evidence that infants found the unfair distribution to be more unexpected than the fair distribution.

#### Test trials

An ANOVA looking at infants’ looking time at test as a function of familiarization and test trial revealed no significant main effects of familiarization, *F*(1, 55) = 1.639, *p* = 0.206, or test event, *F*(1, 55) = 2.608, *p* = 0.112. The interaction between familiarization and test trial was not significant, *F*(1, 55) = 2.016, *p* = 0.162; however, given our analytic plan we proceeded to conduct planned comparisons on looking times to the test events as a function of condition.

**Figure 4:**
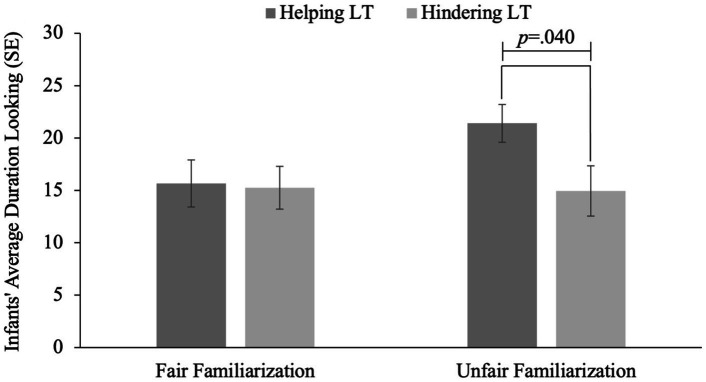
Infants’ average duration looking to helping and hindering following fair and unfair behavior.

An independent samples *t*-test revealed that infants who were familiarized to fair distributions looked equally to the helping (*M* = 15.67, *SE* = 2.25) and the hindering behavior (*M* = 15.25, *SE* = 2.03), *t*(26) = 0.137, *p* = 0.892, *d* = 0.052. In comparison, infants who were familiarized to the unfair distribution looked longer at the helping (*M* = 21.42, *SE* = 1.81) behavior, than the hindering (*M* = 14.96, *SE* = 2.39) behavior, indicating that infants were surprised when the unfair distributor later helped rather than hindered, *t*(26) = 2.157, *p* = 0.040, *d* = 0.815 (Figure 4).

## Discussion

We sought to investigate whether infants use information from one sub-domain to form expectations of how an individual will act in another sub-domain, whether they do so in a bi-directional manner, and whether the strength of their tendency to do so varies according to the source sub-domain. In Experiment 1, we found that after infants were familiarized to an agent who helped another agent in obtaining her goal, infants looked longer to that individual perform unfair versus fair resource distributions. In contrast, after seeing an agent hinder another agent, infants suspend expectations for equal resource distributions for that agent. Together, these results provide a conceptual replication of Surian et al. (2018) and extend these results to events that involve human actors (rather than animated agents) and novel exemplars of helping/hindering behavior. Additionally, whereas Surian et al. (2018) used a 2:0 versus 1:1 distribution, our experiments featured a 5:1 versus 3:3 distribution. Distributions that feature 2:0 outcomes may conflate social exclusion (since one recipient is not included in the exchange at all) with unfairness; in our experiment, by ensuring that each recipient was involved in the exchange but one recipient received more resources than another, our experiments deconflated unfairness from social exclusion, and demonstrate that infants use hindering behavior to make inferences to guide their expectations about fairness/unfairness *per se*.

In Experiment 2 infants were familiarized with a fair or unfair distribution and then saw helping or hindering on test. On test trials, infants’ attention to the helping and hindering varied based on their familiarization type. Infants looked equally at helping or hindering actions performed following an agent’s fair distribution. In contrast, infants looked longer at helping than hindering after seeing an agent perform an unfair distribution. These results suggest that whereas prior fair behavior has no impact on infants’ helping and hindering expectations, unfair behavior leads infants to believe that the individual will subsequently hinder, rather than help, another agent achieve her goal.

Across our two experiments, we found differential effects of the initial event on infants’ subsequent reactions to a second morally valenced behavior. Specifically, in Experiment 1, witnessing helping (a positive moral behavior) led infants to expect subsequent fairness (versus unfairness) whereas witnessing hindering (a negative moral behavior) led infants to have no expectations for fair or unfair behavior. In Experiment 2, witnessing fairness (a positive moral behavior) led to no expectations for helping versus hindering, whereas witnessing unfairness (a negative moral behavior) led infants to expect subsequent hindering behavior (vs. helping behavior). This pattern of findings is consistent with two distinct possibilities.

The first possibility is that infants’ fairness/unfairness and help/hinder expectations are differentially affected by the valence of prior moral behavior; from this perspective, witnessing helping led infants to form subsequent prosocial expectations whereas as witnessing unfairness led to subsequent anti-social expectations. Another way to put this is that infants may see hindering as more influential than helping for forming specific subsequent expectations in the fair/unfair sub-domain, and, conversely, that infants may see unfairness as more influential than fairness in forming subsequent expectations for helping/hindering. Other work has revealed differential effects of positive and negative information on competence versus morality judgments, showing that positive information more strongly influences competence judgments and negative information more strongly influences moral judgments (Wojciszke et al., 1993; Trafimow, 2001). It is possible that such differential effects of initial positive versus negative information also exist between sub-domains of morality, particularly since many prior studies manipulate morality primarily through harm/care scenarios (i.e., Wojciszke et al., 1993).

A second possible interpretation of our findings is that the results reflect initial differences in baseline expectations across the two sub-domains: much work has found that infants have baseline expectations for fair over unfair behavior (Geraci and Surian, 2011; Schmidt and Sommerville, 2011; Sloane et al., 2012; Ziv and Sommerville, 2017; Sommerville and Enright, 2018), whereas no particular expectations for whether a given individual will be helpful or hindering (Hamlin et al., 2007; Fawcett and Liszkowski, 2012; Hamlin, 2013; Margoni and Surian, 2018; Tan and Hamlin, 2022). Although infants did not look significantly longer at the unfair distribution (versus fair) during familiarization in Experiment 1, as might be expected by prior work (Geraci and Surian, 2011; Schmidt and Sommerville, 2011; Sloane et al., 2012; Burns and Sommerville, 2014), infants’ attention declined to the fair distribution across familiarization trials but not the unfair familiarization (whereas this was not true for either helping or hindering behavior during familiarization trials; in both cases, infants’ attention declined significantly). Thus, it is possible that in the current experiment infants may show a weak baseline expectation for fair distributions which may be due to either the difference in the task structure (i.e., no preview of familiarization trial, recipients’ faces not visible to infant) or the fact that testing occurred via Zoom. From this perspective, across both experiments witnessing initial prosocial behavior (i.e., helping, fairness) has no impact on baseline expectations for subsequent prosocial or anti-social behavior, whereas initial antisocial behavior (unfairness, hindering) shifts baseline expectations. Critically, this perspective is in keeping with work from adults showing stronger effects of negative moral information on moral trait inferences than positive information (Wojciszke et al., 1993); however, our current findings do not allow us to distinguish these alternatives.

Regardless, both interpretations are consistent with the broader claim that in the second year of life infants are capable of generalization across moral sub-domains. But are there qualitatively distinct interpretations possible that do not necessarily involve moral reasoning on the infants’ part at all? One possibility is that infants may have construed the protagonist’s behavior in the familiarization trials in terms of whether it facilitates another agents’ goals or disrupts it; in other words, infants may represent these events solely in terms of the protagonists’ role in an inter-personal interaction. Agents that help and enact fair distributions act as goal facilitators (by helping another agent get their desired toy or get an optimal number of resources), whereas those that hinder and enact unfair distributions (by hindering another agent’s access to a desired toy or minimizing the number of resources obtained) disrupt others’ goals. It is possible that infants respond on test based on the role that the protagonist adopts as either a goal facilitator or goal disrupter, and whether it is consistent with their prior role (i.e., looking longer when a goal facilitator becomes a goal disruptor and vice versa). However, it seems less likely than other interpretations given the asymmetry we observed in our data; thus, we favor the broader conclusion that our data support the interpretation that infants generalize across moral sub-domains while recognizing the exact way that they do so requires further study.

While our findings suggest a bidirectional tendency to generalize from moral norm violations from help/harm to fairness and vice versa, a descriptive characterization of the effect sizes across studies indicates that infants generalize more strongly from hindering behavior to unfairness (*d* = 1.067) than they do from unfair behavior to hindering (*d* = 0.815). Coupled with the fact that infants show a sensitivity to help/harm prior to when they show a sensitivity to fairness/unfairness (Sommerville, 2022), and the fact that individuals tend to see hindering as more egregious than unfairness (Yucel et al., 2022), these findings raise the possibility that the degree to which infants will generalize from a given moral behavior may vary according to the severity of that behavior. Future work can directly test this idea.

There are several possible limitations of our work that bear consideration. One possible limitation is that we only used single examples of helping/hindering actions, and fair/unfair distributions. Future work should confirm whether our findings generalize more broadly to other exemplars of these behaviors. Another limitation of our work could be that our sample did not formally test infants younger than 14-months, and as such we cannot speak to the developmental origins (or lack thereof) of infants’ generalization. However, preliminary findings in our lab testing 12- and 13- month-old infants provide no evidence that infants of this age generalize across moral sub-domains. Assuming this developmental transition is replicated, one possibility is that the transition to generalization reflects a domain-general change in infants’ generalization abilities. Alternately, this transition may be better explained by a domain-specific shift in behavioral generalization that is spurred by infants’ increasing language comprehension and exposure to common moral trait terms (i.e., nice, mean) provided by parents and caregivers. Young children show evidence of using moral trait terms between 19- and 22-months (Bloom et al., 1975; Fenson et al., 1994), and by 30-months, they even begin to apply these terms to morally relevant contexts (Snow, 1987). Given that infants’ language comprehension frequently exceeds their production, future studies can empirically test the role of exposure to common trait terms in the face of both adherence to and transgressions of moral norms to determine their role in infants’ generalization across moral sub-domains.

In closing, here we demonstrate that infants possess an ability to generalize across moral sub-domains of fairness and help/harm. Given findings that young children show similar patterns of generalization across moral sub-domains (Gill et al., under review, see footnote 1), these results point to striking commonalities between older children and infants’ tendency to rapidly generalize from moral transgressions. These results open the door to a more fulsome investigation into infants’ tendency to engage in behavioral generalizations, and raise important directions for future work, including the age of emergence of this tendency, the scope of generalization, and the underlying mechanisms supporting these generalizations.

## Data availability statement

The raw data supporting the conclusions of this article will be made available by the authors, without undue reservation.

## Ethics statement

The studies involving human participants were reviewed and approved by University of Toronto (U of T) Research Ethics Board (REB). Written informed consent to participate in this study was provided by the participants’ legal guardian/next of kin.

## Author contributions

IG and JS conceptualized the study, performed the formal analyses, developed and designed the methodology, were responsible for project administration, and reviewed and edited the final draft. IG was responsible for data collection and investigation, wrote the original draft, and prepared and presented the work throughout data collection under JS supervision. JS acquired funding for the project. All authors contributed to the article and approved the submitted version.

## Funding

This research was supported by a SSHRC Insight Grant 435–2022-0118 and by a grant from the John Templeton Foundation. Both grants were awarded to JS.

## Conflict of interest

The authors declare that the research was conducted in the absence of any commercial or financial relationships that could be construed as a potential conflict of interest.

## Publisher’s note

All claims expressed in this article are solely those of the authors and do not necessarily represent those of their affiliated organizations, or those of the publisher, the editors and the reviewers. Any product that may be evaluated in this article, or claim that may be made by its manufacturer, is not guaranteed or endorsed by the publisher.

## Footnotes

^1^Gill, I. K., Curtin, A., and Sommerville, J. A. (under review). Children’s inferences of moral character across different moral sub-domains. *Dev. Psychol*.

^2^Parent and child demographic information were acquired *post hoc*. As such, response rates were low.

^3^The experimenter verified that infants watched each familiarization trial in entirety. Though rare, if infants looked away during the critical period of the trial, the trial was restarted; this was recorded for 2 participants across both experiments.

^4^Though we aimed for 50% of the sample for reliability coding, due to lack of parental consent and Zoom errors, we did not have videos for all participants.

^5^One participant did not look at Familiarization Trial 4 and the results are the same with the participant included or excluded.

^6^Parent and child demographic information were acquired post-hoc. As such, response rates were low.

^7^Excluding the parents who declined consent to have their testing session recorded.
